# The effects of pomegranate extract on normal adult rat kidney: A stereological study

**Published:** 2016-03-15

**Authors:** Esrafil Mansouri, John Basgen, Sadegh Saremy

**Affiliations:** 1 Department of Anatomical Sciences, Cellular and Molecular Research Center, Faculty of Medicine, Jundishapur University of Medical Sciences, Ahvaz, Iran;; 2 Life Sciences Institute, Charles R. Drew University of Medicine and Science, Los Angeles, USA.

**Keywords:** Kidney, Pomegranate, Rat, Stereology

## Abstract

Pomegranate *(Punica granatum L.)* has been used widely in the traditional medicine of various civilizations for more than 5000 years. The pomegranate tree has several parts; each part has useful medicinal effects. Previous studies have demonstrated the antibacterial, antioxidant, and anti-inflammatory properties of pomegranate. The aim of the present study was to determine whether administration of pomegranate extract could result in morphometric changes in the kidneys of rats. Eighteen male rats (180-200 g) were divided into three groups that received either: G1, distilled water; G2, 250 mg kg^-1^ pomegranate extract; and G3, 500 mg kg^-1 ^pomegranate extract via oral gavages daily for eight weeks. At the end of eight weeks, the rats were euthanized and their kidneys were removed and processed for morphometric analyses. In rats received pomegranate extract, the kidney weight, kidney weight/body weight ratio, cortex v/lume and glomerular volume were increased (*p* < 0.05), while, medulla volume and the number of glomeruli per kidney did not change. No pathological lesions were observed in the kidney. Therefore, pomegranate hydro-alcoholic extract at doses of 250 and 500 (mg kg^-1^) increased the volume of some parts of the kidney; however, it did not cause any pathological changes in the kidney.

## Introduction

The pomegranate plant, *Punica granatum L*, is widely distributed in Middle Eastern countries, including Iran.^1^ Pomegranate is one of the most widely known traditional edible plants. It is mentioned in the Quran, the Bible, the Torah, and the Babylonian Talmud as the ‘Food of Gods’ symbolizing abundance, fruition and successfulness.^2^ Since ancient times, the pomegranate has been used in the traditional medicine of various civilizations as a “curative food” for fever, ulcers, diarrhea, acidosis, dysentery, hemorrhage, microbial infections, parasites and respiratory disorders.^3^^,^^4^ The pomegranate tree has several useful medicinal parts: seed, juice, peel, leaf, flower and root bark. Each of these has been associated with some beneficial effects on health, such as preventing cancer and arterio-sclerosis and lowering high cholesterol. The chemical composition of the pomegranate depends on its growing area, weather, the phase of fruit maturity, methods of cultivation and processing steps.^5^^,^^6^ The useful effects of pomegranate are related to its extensive spectrum of phytochemicals, such as tannins, alkaloids, and dyes.^7^^,^^8^ The polyphenols are the major class of phytochemicals which found in pomegranate, including hydrolysable ellagitannins and anthocyanins. Ellagitannins, found in the outer compartment of the fruit, are the main source for the antioxidant properties of pomegranate extract. It has been demonstrated that ellagitannins and punicalagin anomers A and B are responsible for over 50% of the antioxidant activity of pomegranate juice.^9^ There is little information regarding the biodistribution, bioavailability, absorption and metabolism of bioactive ingredients of pomegranate and other fruits, but probably they all have similar mechanisms.^10^ No deleterious side effects have been reported after consuming pomegranate and its compounds. Moreover, experimental research animals have failed to demonstrate any toxicity when given a normal dose.^11^ In addition, administration of pomegranate juice didn’t show toxicity in histopathological evaluations.^12^ Recently, a number of researchers have tried to determine the mechanisms responsible for the medicinal effects of pomegranate. These previous studies have concentrated primarily on the antibacterial, antioxidant, and anti-inflammatory properties of pomegranate.^13^^-^^15^ Despite the many studies on the pomegranate and its effects on various systems,^16^ a quantitative microscopic study of the effects on the kidney has not been reported. 

The present study designed to study the effects of pomegranate extract on the structure of rat kidney.

## Materials and Methods

**Pomegranate extract.** Pomegranate fruit (Farabi Herbarium number GUE 7321) was purchased from the local market and then dried at room temperature away from sunlight for a week. After drying, all components of the fruit (seed, peel) were made into powder with an electric mill. Next, 500 g of the powder were poured into a flask with two liters of 70% ethanol. The mixture was stirred intermittently and after 72 hr, filtered using a funnel blocked with cotton wool. The filtered solution was placed in a flat container and allowed to dry at ambient temperature. The resulting dry extract was stored at 4 ˚C until used.^17^ Immediately before use, the extract was mixed with 1mL of distilled water. 

**Experimental animals. **Eighteen male Wistar rats (180 to 200 g) were kept at the central animal house of Ahvaz Jundishapur University of Medical Science. All rats were housed in cages with 12/12 hr light/dark cycle at 21 ± 2 ˚C. All animal experiments were carried out in accordance with Ahvaz Jundishapur University Ethical Committee (AJUEC). Rats were divided into three groups of six rats each: G1) served as the vehicle control group and received 1 mL of distilled water by gavage once a day for eight weeks; G2 and G3) these groups received 1 mL of distilled water that contained enough extract to make the doses equal to 250 and 500 mg kg^-1 ^of body weight, respectively. At the end of eight weeks,^18^ rats were sacrificed under ether anesthesia and the right kidney was cleaned completely of perirenal fat and connective tissue as well as the renal pelvis. The kidneys were fixed in 10% formalin for three days. 

**Fig. 1:**
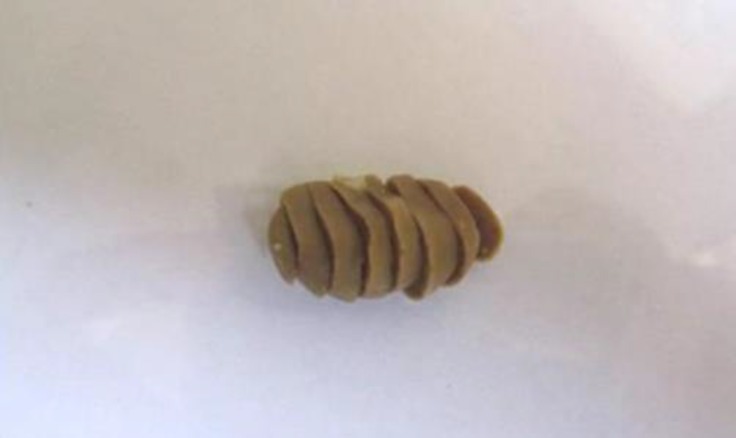
Kidney slabs cut in parallel sections in 2 mm thickness sequentially. These slabs are ready for superimposition of the grid

**Fig. 2:**
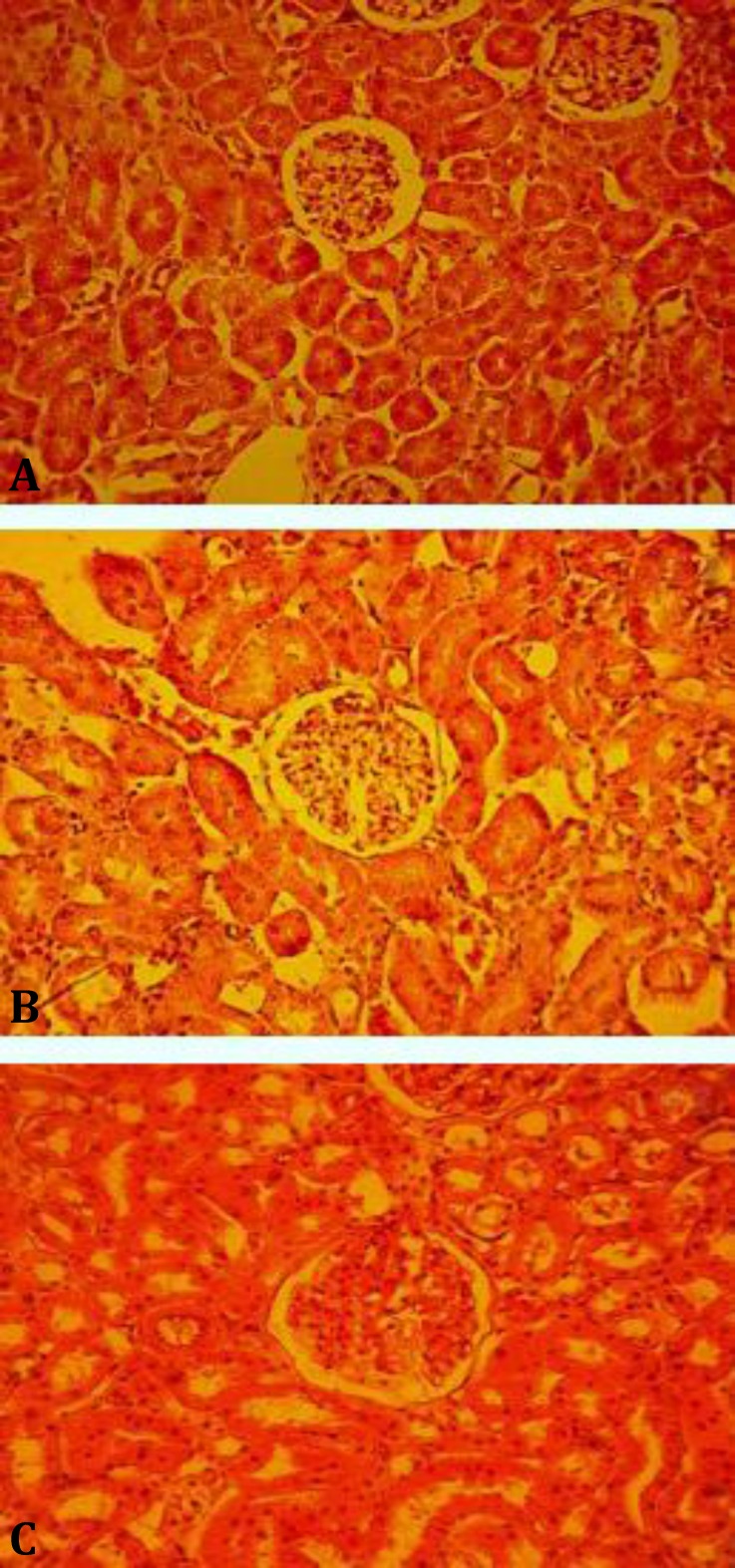
Microscopic slides of kidney in different groups; **A)** Control group, received 1 mL of distilled water; **B)** G2: received 250 mg kg^-1^ extract; and **C)** G3: received 500 mg kg^-1^ extract, (H & E, 20×).

**Tissue preparation.** The fixed-kidneys were cut perpendicularly to the major axis into a series of slabs with a thickness of 2 mm beginning at a random position within the first 2 mm of the kidney. Eight to ten slabs were obtained from each kidney (Fig. 1). Since the first slab has a natural (not cut) surface, it was not used.^19^ The other slabs were dehydrated through a series of ethanol and embedded in paraffin. Five micrometer thick sections were cut using a rotary microtome and then, sections stained with hematoxylin and eosin (H & E), (Fig. 2). For determination of glomerular number, a dissector was made using a pair of sections a known distance apart.^20^ In this experiment the first technically good section (the reference section) and the third section after it (the look-up section) were saved from each kidney slab forming 20 µm dissectors. 

**Imaging. **A pair of 1000X-1 microprojectors (Ken-A-Vision Inc., Kansas City, USA) were used to project images of the kidney sections onto a desktop at a magnification of 20×. Projections have done in a dark room.

**Fig. 3:**
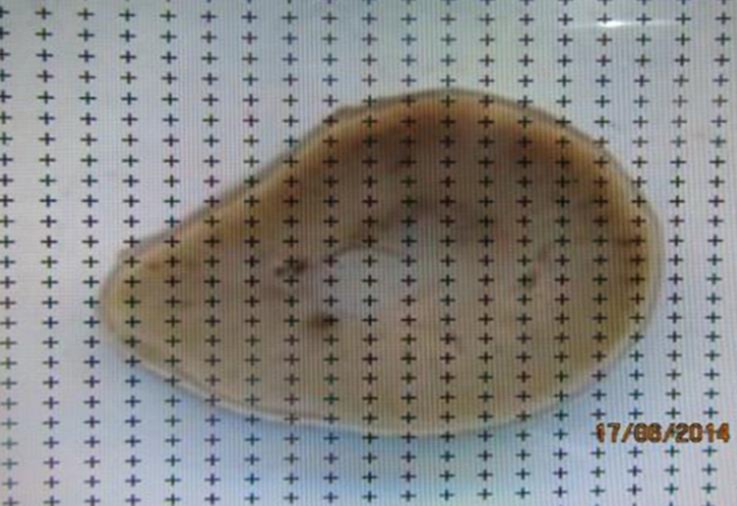
Superimposition of the point grid covering the slice and estimation of the area of the slice using a transparent point grid

**Total** **kidney, cortex, and** **medulla** **volumes** **determination. **Kidney volume was measured using the Cavalieri principle.^21^ Briefly, before the kidney slabs embedded in paraffin, a transparent test point system (a grid) superimposed randomly on the right cut surface of the kidney slabs and the grid points that “hit” the kidney counted (Fig. 3). Kidney volume was estimated using the formula:

$V_{\mathrm{kidney}}({\mathrm{mm}}^{3})=\frac{\sum P\times (a/p)\times t}{M^{2}}$

where, *∑P* denotes the number of points counted on kidney, (*a/p*) represents the area associated with each grid point (4 mm^2^), *t* is the mean slab thickness (20 mm) and *M* (1×), is the linear magnification.^22^

To estimate the cortex and medulla volumes, we selected the first section in each paired sections (the reference sections). Its image was made by a micro-projector on the desk. Then, the image randomly covered with a transparent grid composed of points. The points that “hit” the cortex or medulla counted separately for all reference sections from a kidney. Points that had hit the kidney but not cortex or medulla were also counted. Volume density of each of the two kidney components was determined using the formula: 

$V_{v}(\mathrm{component}/\mathrm{kidney})=\frac{\sum P(\mathrm{component})}{\sum P(\mathrm{kidney})}$

where, *∑P* (component) indicates the number of points hitting the component, either cortex or medulla, and *∑P* (kidney) is the number of points hitting the kidney. 

Finally, the volumes of cortex and medulla were determined by multiplying the volume density of each component by the volume of kidney:

*V*_medulla_ *(* *mm* ^3^ *) = V*_v_ *(medulla/kidney) × V*_kidney_

*V*_cortex_ *(* *mm* ^3^ *) = V*_v_ *(cortex/kidney) × V*_kidney_

The magnification of the projected images was 20×. Volume density of glomeruli per cortex (*V*_v_; glomerulus/ cortex) was measured using approximately 15 random microscopic fields from each reference slide. A grid of points superimposed over the cortex and the number of points hitting the glomeruli and the number of points hitting cortex counted. Then, *V*_v_ (glomerulus/cortex) was calculated using the formula:

$V_{v}(\mathrm{glomerulus}/\mathrm{cortex})=\frac{\sum P(\mathrm{glomerulus})}{\sum P(\mathrm{cortex})}$

where, *∑P* (glomerulus), is the number of points falling on the glomeruli and *∑P* (cortex) is the number of points that fell on cortex.

Total glomerular volume was estimated using the following formula.^23^^,^^24^ Magnification of the projected images was 20×.

*V*_total glomeruli_ *(* *mm* ^3^ *) = V*_v_ *(glomeruli/cortex) × V*_cortex_

**Glomerular number determination** **. **For estimating the total number of glomeruli by the physical dissector method, two microprojectors were used simultaneously.

**Fig. 4:**
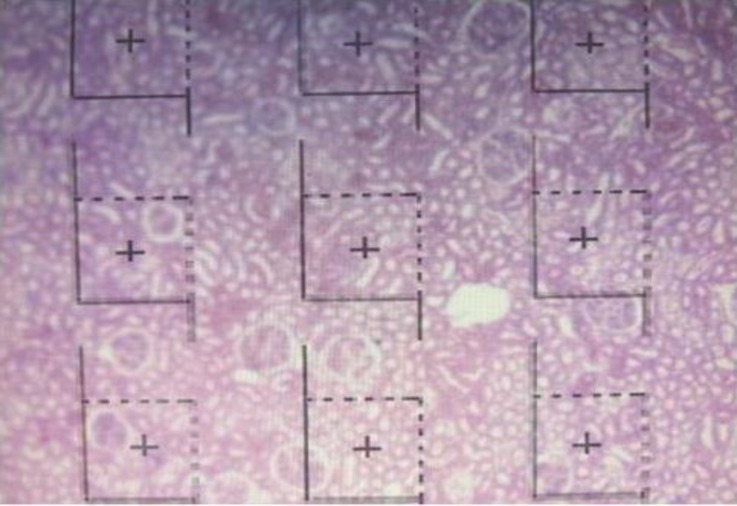
Superimposition of the dissector frame covering the tissue slides and estimation of total number of glomeruli

The images of the reference and look-up sections were projected on to the desktop at the same time. Using an unbiased 2-D counting frame,^25^ approximately 50 visual fields in each kidney were selected by systematic uniform random sampling (SUR) method and glomeruli were counted (Fig. 4).

In this method, the glomeruli that were in the reference section and within the unbiased counting frame but were not in the look-up sections were counted. Each of the dissector frames had a central point; if this point placed in the reference space, the volume of related dissector added. Using the following formula, numerical density of the glomeruli was calculated:

$V_{v}(\mathrm{glomeruli}/\mathrm{cortex})=\frac{{\sum Q}^{-}}{\sum P\times \mathrm{Area}\times \mathrm{Height}}$

where, *∑Q*^–^, is the number of the glomeruli seen in the reference section but not in the look-up section, *∑P*, the number of points and also frames that crossed the reference space, *Height* is the height of the dissector, and *Area* is the frame area divided by the square of the magnification.

Finally, to determine total number of the glomeruli in a kidney, the following formula was used.^26^^,^^27^

*N*_total glomeruli_ *= N*_v_ *(glomeruli/cortex)* *×* *V*_cortex_

**Statistical Analysis** **.** Data expressed as the mean ± SD. One-way ANOVA by SPSS (Version 18; SPSS Inc., Chicago, USA) was used for statistical analysis followed by Tukey’s post hoc test. A *p-*value less than 0.05 was assumed as statistically significant.

## Results

**Kidney weights and kidney weight/body weight ratio.** There was a significant increase (*p* < 0.05) in kidney weights as well as kidney weight to body weight ratio in the two groups received the pomegranate extract compared to the control group (Table 1). 

**Total kidney, cortex and medulla volumes.** Analysis of data revealed that kidney and cortex volumes were increased (*p* < 0.05) in both groups received the extract compared to the control group. The medulla volume was not significantly different in rats that received pomegranate extract compared with the control group (Table 2). An average of 1499 points counted on slabs of kidney, 1671 points on cortex, and 861 points on medulla per rat.

**Table 1:** Comparison of kidney weight/body weight ratio and kidney weight of rats between groups after administration of pomegranate hydro-alcoholic extracts for eight weeks. Data are expressed as mean ± SD

**Groups**	**Kidney weight/body weight (mg per g)**	**Kidney weight (mg)**
**G1**	3.18 ± 0.24	864.50 ± 46.34
**G2**	3.73 ± 0.13^*^	1031.33 ± 40.32^*^
**G3**	3.57 ± 0.24^*^	981.16 ± 66.88^*^

G1 (control group): received distilled water; G2: received extract (250 mg kg^-1^); and G3: received extract (500 mg kg^-1^). * indicates significant difference compared with the G1 group (*p *< 0.05).

**Glomerular volume.** Glomerular volume significantly increased (*p* < 0.05) in the two groups received pomegranate extract in comparison with the control group (Table 2). The average number of points per rat hitting cortex was 1671 and hitting glomeruli was 67 glomerular numbers. The total number of glomeruli did not show any significant differences between groups (Table 2). An average of 113 glomeruli counted per rat.

**Table 2:** Comparison of kidney, cortex, medulla and glomerular volumes (mm^3^) and total number of glomeruli between groups after administration of pomegranate hydro-alcoholic extracts for eight weeks. Data are expressed as mean ± SD

**Groups**	**Kidney volume** **(×10 mm** ^3^ **)**	**Cortex volume** **(×10 mm** ^3^ **)**	**Medulla volume** **(×10 mm** ^3^ **)**	**Glomeruli volume** **(×10 mm** ^3^ **)**	**Total number** **of glomeruli**
**G1**	663.50 ± 30.85	446.33 ± 24.01	180.33 ± 10.59	15.66 ± 3.01	29363.17± 284.68
**G2**	783.00 ± 24.57*	565.00 ± 27.60*	194.00 ± 6.72	23.50 ± 4.5*	29041.67 ± 283.92
**G3**	764.16 ± 47.39*	559.16 ± 31.79*	191.83 ± 12.38	22.16 ± 5.03*	29087.50 ± 289.00

G1 (control) received distilled water; G2 received extract (250 mg kg^-1^); and G3 received extract (500 mg kg^-1^).

* indicates significant difference compared with the G1 group (*p *< 0.05).

## Discussion

Plants have been used for medicinal purposes for more than 5,000 years. In recent years, researchers have begun to study the molecular mechanisms associated with the beneficial effects of medicinal plants. Interest in polyphenols has grown considerably because of their high capacity to trap free radicals associated with different diseases. Phenols and flavonoids which have antioxidant activities have been shown to be very important plant constituents. The plant phenolics are commonly present in fruits, vegetables, leaves, nuts, seeds, barks, roots and in other plant parts.^28^ Pomegranate is an important source of vitamin C.^18^ The antioxidant and free radical scavenging activities of pomegranate phenolic compounds and vitamin C have been reported.^29^^,^^30^ Our study showed a significant increase in kidney weight/body weight ratio in groups which received the extract. This finding is similar to a previous study.^31^ In addition, in the present study designed-based stereology was used and an increase in kidney weight, volumes of total kidney, cortex and glomerulus and subsequently volume density of cortex and glomeruli was found. 

The results of this study are in accordance of previous reports.^18^^,^^32^ It may be due to which portions of kidney affected by flavonoid. The kidney is composed of cortex and medulla. The major effects were on the cortex. The cortex consists of several structures but its main parts are the tubules and glomeruli.^33^ From these results, it appears that pomegranate extract has affected the glomeruli in the cortex more than other parts. One could suggest that, the flavonoids - containing compounds might cause vasodilation and blood flow increase in the kidney.^34^ In addition, it has been reported that polyphenolic compounds could elevate nitric oxides that increase blood flow.^35^ This increased blood flow can also influence glomeruli and increases the size of the glomeruli that could be a main factor in increasing the weight, volume density and volume of cortex, volume density of glomeruli and total volume of glomeruli. We did not find significant differences in volume and volume density of medulla in this study. These findings are in agreement with report of a previous study.^36^ The data obtained from this study indicated that, tubules of kidney were less affected by pomegranate extract. Since the medulla is composed mainly of tubules, thus in this part of kidney no significant difference was found. We observed a slight increase in size of the medulla, which may be due to the increased volume of blood vessels due to increased blood flow or due to increase of interstitial tissue and urinary tubules in this part of kidney that should be evaluated for confirmation. As results indicated, there was no significant difference in numerical density and total number of glomeruli after administration of pomegranate extract. Side effects after consuming pomegranate and pomegranate extract in different doses have not been reported in previous studies.^11^^,^^12^ Thus, the present study is similar to previous studies.^31^

In conclusion, the results of present study suggest that, oral administration of pomegranate hydro-alcoholic extract at doses of 250 and 500 (mg kg^-1^) increases the volumes of kidney, cortex and glomeruli but did not make a difference in the medulla in rats.
